# Bone Graft Expansion in Cranioplasty Using a Split-Bone Technique

**DOI:** 10.7759/cureus.84790

**Published:** 2025-05-25

**Authors:** Norris C Talbot, Carlie Proctor, Patrick Luther, Michael Folse, Nimer Adeeb, Michael P Minamyer, Jamie Toms

**Affiliations:** 1 School of Medicine, Louisiana State University Health Sciences Center, Shreveport, USA; 2 Neurosurgery, LSU Health Shreveport, Shreveport, USA; 3 Anesthesiology, Louisiana State University Health Sciences Center, Shreveport, USA; 4 Neurosurgery, Louisiana State University Health Sciences Center, Shreveport, USA

**Keywords:** cerebral edema, craniectomy, cranioplasty, craniotomy, neurocritical care

## Abstract

Decompressive hemicraniectomy (DHC) is performed in emergent cases of uncontrollable intracranial hypertension in which noninvasive procedures or medications are not able to safely maintain pressure within the cranium, increasing the risk of morbidity and mortality. The native bone flap is then replaced, a procedure referred to as cranioplasty, nearly three to six months after injury to allow time for brain relaxation. However, in cases with persistent cerebral edema at the time of cranioplasty, techniques are often applied intraoperatively, including mannitol, external ventricular drain, or lumbar drain placement. To avoid the risks of delaying the procedure or drain placement, we demonstrate a novel technique of splitting the bone flap to adequately increase the size and flexibility. Three patients with a mean age of 44 underwent this novel technique during cranioplasty due to persistent brain edema following a DHC. The new operative technique was successfully performed to compensate for the lingering edema, and all three patients were monitored postoperatively, showing no complications. In this study we demonstrate a new technique to alter bone flap size and flexibility during cranioplasty cases with persistent brain edema, avoiding the need for invasive drain placement. All patients experienced no complications or new cranial/skull defects postoperatively.

## Introduction

Decompressive hemicraniectomy (DHC) is performed to acutely relieve intracranial hypertension, which could be a result of traumatic brain injury, malignant stroke, or intracranial bleeding, that has not responded to less invasive treatment [1,2]. A multitude of agents can be utilized to attempt to lower intracranial pressure such as osmotic agents, shunts, 3% hypertonic saline, carbonic anhydrase inhibitor drugs, lumbar punctures, and external ventricular drains [3]. However, some cases that are either too emergent or resistant to less invasive treatment result in a DHC to control the intracranial pressure and allow more space for the brain to swell. In those cases, the utilization of DHC has been linked to reducing mortality and potentially improving the overall functional outcome [4,5].

Certain trials have shown the benefits of craniectomy within emergencies to improve mortality results and minimize motor deficits. The Decompressive Craniectomy in Malignant Middle Cerebral Artery Infarction (DECIMAL Trial) reported that a decompressive craniectomy, when performed early, can increase by over half the amount of patients with moderate disability while reducing mortality by more than half when compared to medical therapy [6]. The DESTINY trial found that hemicraniectomy reduced mortality in patients with large ischemic stroke, although it could not show statistical superiority because the trial was terminated early due to results published in other trials [7]. The DESTINY II trial was able to show hemicraniectomy, in patients over age 61 with malignant middle cerebral artery (MCA) occlusion, increased survival without disability [8]. The Hemicraniectomy After Middle Cerebral Artery Infarction with Life-threatening Edema Trial (HAMLET) showed that surgical decompression can reduce case fatality and poor outcomes if done within 48 hours but no benefit to functional outcome if delayed to 96 hours or later past stroke onset [9]. The Hemicraniectomy and Durotomy Upon Deterioration From Infarction-Related Swelling Trial (HeADDFIRST) showed that in many patients, medical management alone can result in lower mortality, possibly due to adherence to a standardized medical therapy protocol in the HeADDFIRST trial [10].

Cranioplasty is recommended to be performed once the brain has relaxed enough for the skull flap to be replaced, typically around three to six months post-DHC. While the optimal timing for cranioplasty has conflicting findings within the literature, cranioplasty protects the cerebral cortex from detrimental injuries, lowers infection risk, and restores the cosmetic shape of the head [11,12]. Weeks or months after a craniectomy, some patients might develop sinking skin flap syndrome (SSFS) causing orthostatic hypotension, motor deficits, cognitive decline, or seizures [13]. SSFS is treated with cranioplasty [13]. Artificial bone graft materials such as polyetheretherketone (PEEK) and titanium mesh or autologous bone in a native bone graft can all be utilized for cranioplasty. PEEK has primarily arisen as a cranioplasty method to improve success rates and complication rates in the setting of inability to utilize native bone [14]. Although hospital stays and operation times were lower as well for PEEK cranioplasties, autologous or native cranioplasties tend to be the option for cosmetic results, lower costs, and easier patient incorporation [14]. Furthermore, in some patients, PEEK cranioplasty might be chosen to prevent bone resorption, infection, donor site morbidity, and in select cases poor cosmesis [14]. The operative technique in this study is designed for native cranioplasties to allow for more flexibility and positional range when replacing the flap.

During cranioplasty, the bony edges should be well-effaced to avoid the creation of a visible defect in the contour of the skull. Furthermore, appropriate alignment with the skull is important to prevent any extending defects as well as careful management of the temporalis muscle to prevent temporalis wasting [15]. Some techniques have been described such as temporalis muscle wrapping with an autologous dural flap to try and rectify this aesthetic problem [16]. Furthermore, in native cranioplasty bone resorption is a common concern to affect cosmetic appearance postoperatively [17]. Moreover, many patients undergoing cranioplasty must be transferred from facilities, halt anticoagulation medication, or orchestrate care with family. If the decision to operate is maintained, then the patient must undergo general anesthesia and opening of the scalp. As a result, several techniques are often employed to achieve intraoperative brain relaxation and avoid brain compression in cases with persistent brain edema, including IV mannitol, external ventricular drain, lumbar drain, and US needle drainage of superficial fluid collection [3]. Those techniques are associated with several risks, including bleeding, prolonged hospitalization, and infection [18,19]. In this study we present a technique of splitting the bone flap to allow adequate placement and avoid the risk of delaying the procedure and drain placement, ultimately prolonging hospitalization.

## Case presentation

Case 1

A 58-year-old male presented to our center with a right-sided MCA stroke with malignant edema that was found to worsen on subsequent imaging. The patient was prepped for a right-sided frontotemporoparietal DHC to prevent further compression of the brain and, ultimately, herniation. A large trauma flap was planned, and the temporalis was lifted. Burr holes were placed in the frontal and parietal keyholes and a large bone flap was turned. Examination of the brain found it to be non-pulsatile, and meticulous hemostasis was then obtained. A drain was placed underneath the galea on a piece of gel foam and tunneled through the parietal part of the incision and secured. The wound was then closed in layers. The native bone flap was preserved for future re-implantation. The bone flap was transported after craniectomy to be frozen and preserved until the patient underwent the second procedure. This storage provides adequate long-term maintenance and viability of the skull flap until it is placed back. All three cases underwent similar management.

**Figure 1:**
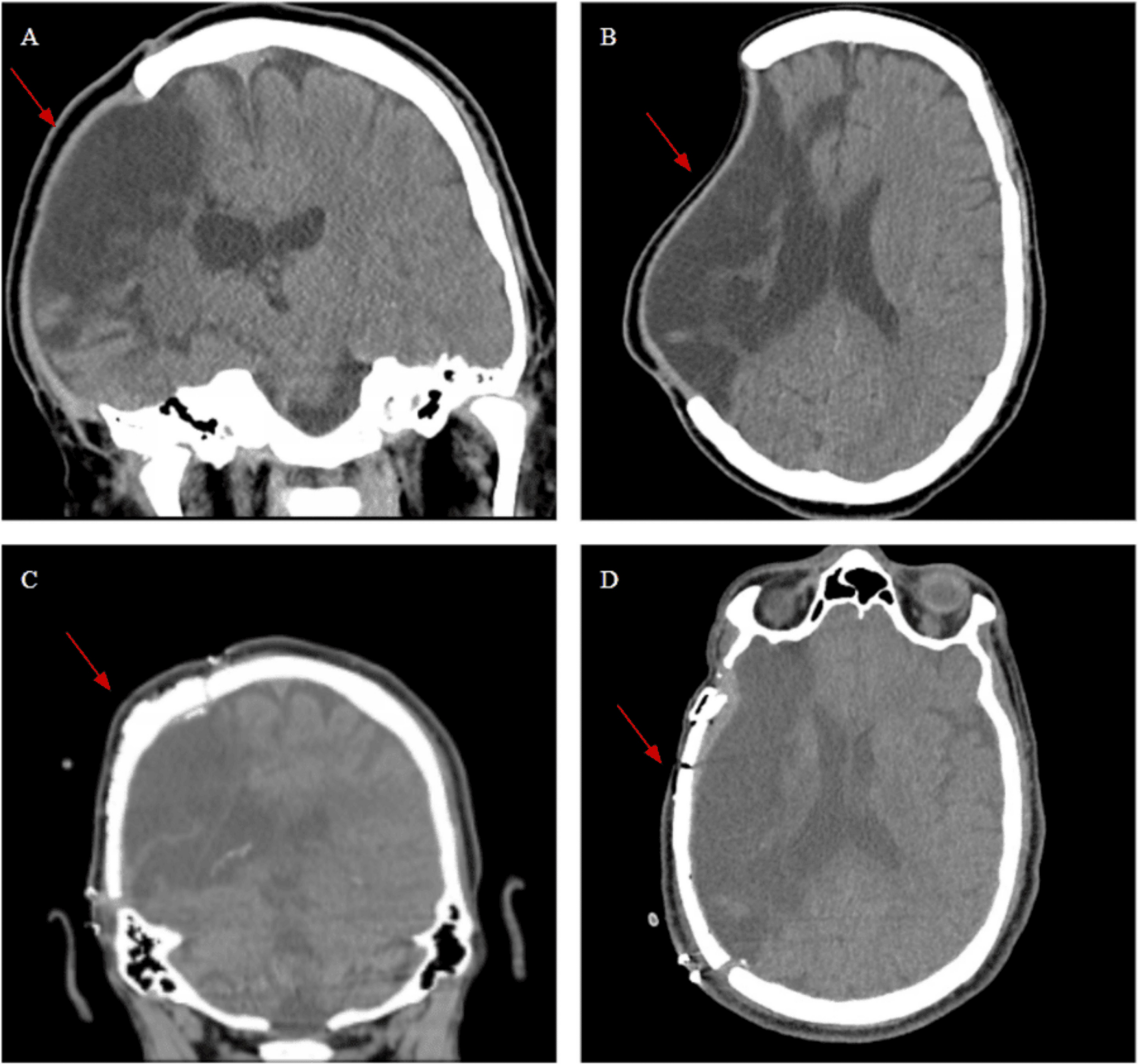
(A, B) Coronal and Axial sections before cranioplasty. (C, D) Coronal and Axial section post-operatively. The arrows point towards the portion of the cranium removed and repaired.

Five months after the initial case, the patient was brought back for repair of the large skull defect. The previous incision was opened, and the bone was freed circumferentially around the skull defect. The bone flap was reapproximated with cranial plating. The brain was found to be slightly swollen, and it was not possible to create a flush placement of the bone flap to the skull. To best reapproximate the flap to the skull given these conditions, the flap was cut in half using a craniotome, and a large cranial mesh was then placed on the center of the halved bone flap to expand the surface area and allow for more room to rest flush with the skull. A subgaleal drain was placed, and the galae was reapproximated using 2-0 Vicryl. The outer layer was closed with staples. Upon follow-up, the wound healed, and no defect was visible due to the implemented technique. A comparison of pre- and postoperative CTs can be seen in Figure 1.

Case 2

A 38-year-old woman presented to our center with mental deterioration over multiple days due to a large left-sided MCA stroke. Imaging showed the large MCA territory infarct with malignant edema, and the patient was taken emergently to the operating room for a left-sided frontotemporoparietal DHC to prevent further compression and herniation. A trauma flap was planned, the temporalis muscle was lifted with the scalp flap, and the bone flap was removed. The brain was tense under the dura. After meticulous hemostasis was achieved with a combination of bone wax, thrombin-soaked Gelfoam, Surgiflow, and bipolar electrocautery, a large Gelfoam layer was placed on the brain. A drain was placed on top of this layer but under the galea, tunneling through the parietal area of the incision to secure it in place.

**Figure 2:**
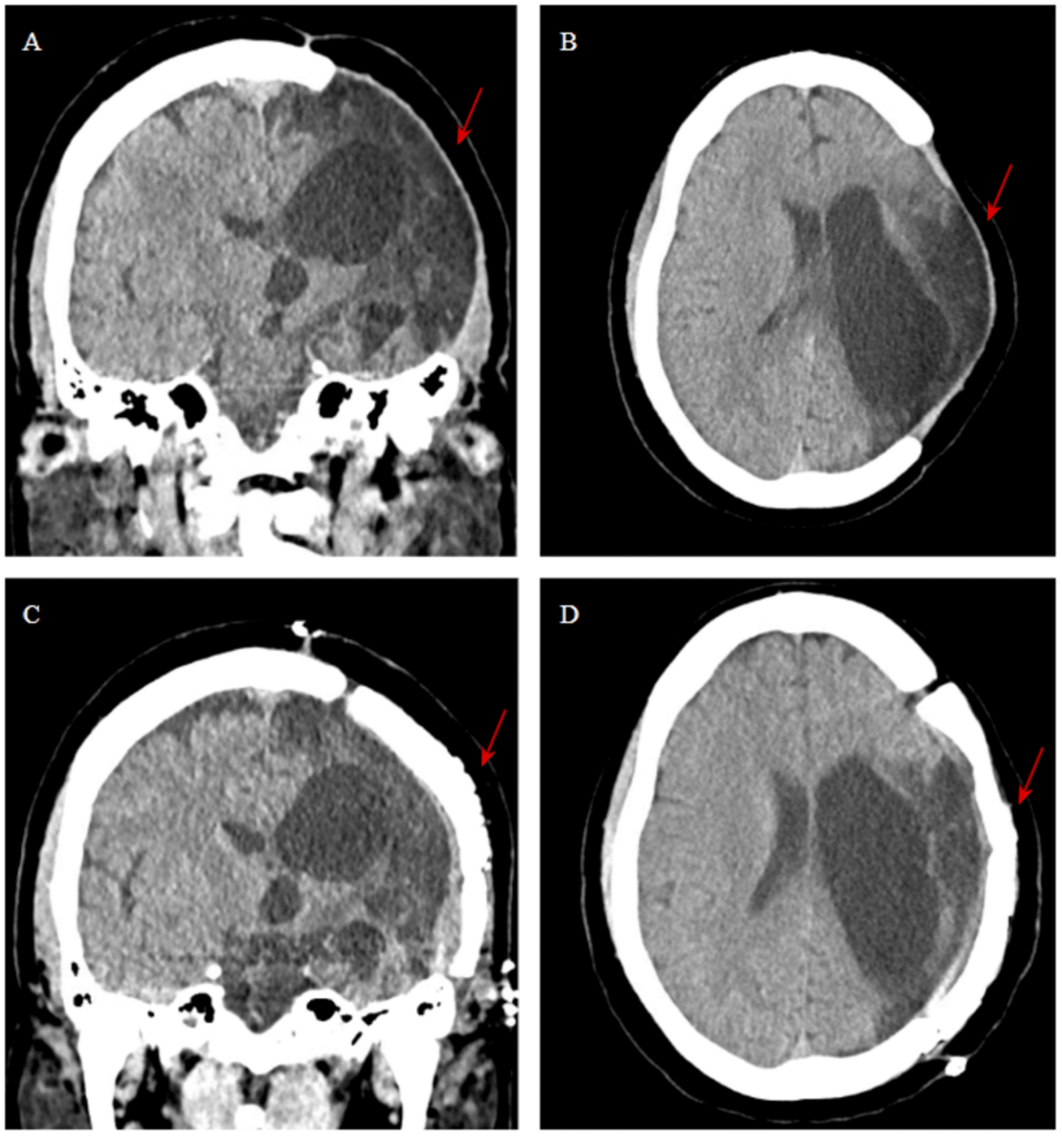
(A, B) Coronal and Axial sections before cranioplasty. (C, D) Coronal and Axial section post-operatively. The arrows point towards the portion of the cranium removed and repaired.

One year later the patient returned to the OR for a left-sided cranioplasty. The previous incision was opened to reveal the known cranial defect. Upon visualization, the brain was edematous, therefore a ventriculostomy catheter was inserted into the left lateral ventricle and 30 mL of CSF was removed. After the removal of the ventricular catheter, the brain was still too swollen for flush bone flap replacement. The bone flap was then divided into two pieces using a craniotome drill, and a large cranial mesh was placed in the center of the two bone flaps to increase the surface area of the flap. The remainder of the bone flap was plated with the cranial plating system and secured onto the skull without difficulty. The galea was reapproximated using 2-0 Vicryl. The outer layer was closed with staples, and the patient recovered well from the surgery. Upon follow-up, the wound healed, and no defect was visible due to the implemented technique. A comparison of pre- and postoperative CTs can be seen in Figure 2.

Case 3

A 51-year-old woman presented to our center with a large intraparenchymal hemorrhage, right-to-left midline shift, right uncal herniation, and diffuse subarachnoid hemorrhage. The patient was taken to the operating room for right-sided DHC with evacuation of a large intracerebral hemorrhage. The scalp was incised, and hemostasis was achieved using electrocautery. The right side of the head was shaven. A large trauma flap was planned. The temporalis muscle was lifted with the scalp flap. Burr holes were placed at the frontal and temporal keyholes, as well as along the convexity. A large bone flap was turned. The dura was then carefully opened in a cruciate manner. There was a large amount of swelling. At this point, we obtained meticulous hemostasis with thrombin-soaked Gelfoam, Surgiflow, and bipolar electrocautery. An ultrasound was used to locate the large intraparenchymal hemorrhage (IPH) and a corticotomy was made over the blood and the large IPH was carefully removed with suction and biopsy forceps. The brain was noted to be less swollen and non-pulsatile at this point. Hemostasis was achieved. At this point, we placed two large pieces of Gelfoam that we manually compressed on top of the brain. A drain was placed underneath the galea and on top of the Gelfoam and tunneled out through the parietal area of the incision, securing the drain in place.

**Figure 3:**
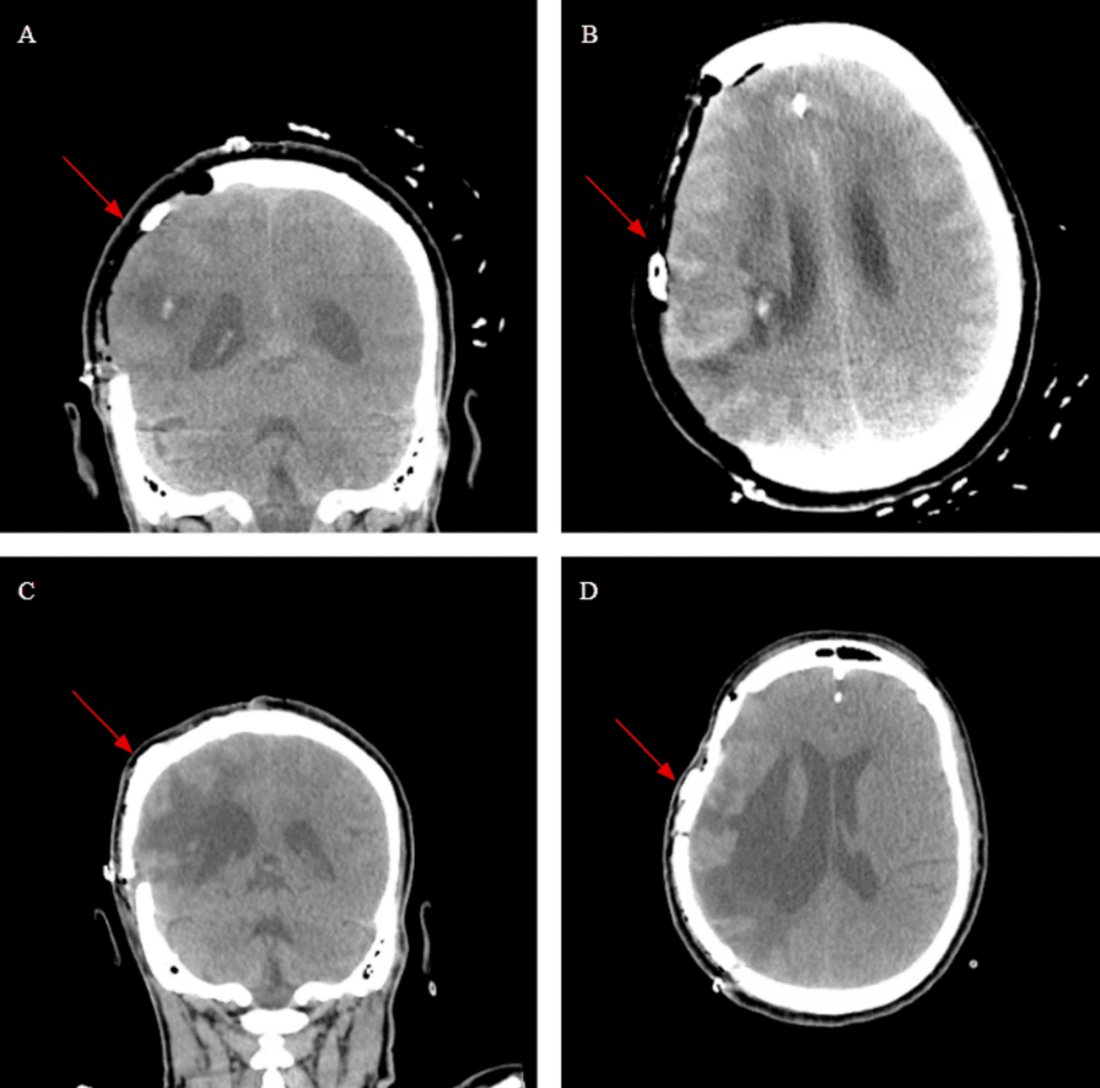
(A, B) Coronal and Axial sections before cranioplasty shortly after the craniectomy. (C, D) Coronal and Axial section post-operatively. The arrows point towards the portion of the cranium removed and repaired.

Eight months later, the patient returned to the OR for right-sided cranioplasty with intracerebral cyst evacuation. The previous incision site was opened and electrocautery was used to reveal the bone underneath. A small opening was made in the brain covering and a needle was guided into the cyst and it was evacuated by removing 40 mL of fluid. Once this had been done and there was a clean bone edge around the entirety of the skull defect, the patient's bone flap was reattached using a cranial plating system. The brain was slightly swollen and did not allow a flush placement of the bone, so the bone was carefully cut in half and a large cranial mesh was used in the center of the bone to expand it and allow for it to rest without pushing on the brain. The galea was reapproximated using 2-0 Vicryl. The outer layer was closed with staples, and the patient recovered well from the surgery. Upon follow-up, the wound healed, and no defect was visible due to the implemented technique. A comparison of pre- and postoperative CTs can be seen in Figure 3.

**Figure 4:**
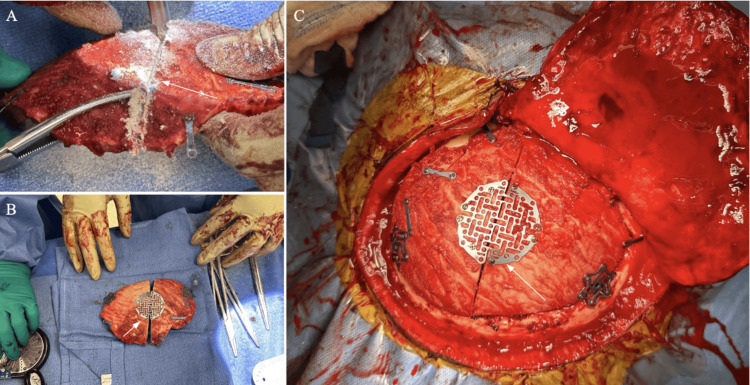
In situ operative viewing of second case. (A) Creating incision along the midline for splitting of the bone flap was done for all three cases. (B) Visualization of extended bone flap before reapproximation. (C) Reapproximated bone flap with graph extension. The arrows show the portion of the flap adjusted to accommodate excess swelling.

Patients with DHC are scheduled for cranioplasty at or after three months from the injury. A CT of the head is performed in the clinic when planning the surgery to assess the status of brain parenchyma, ventricles, and presence of subdural fluid collection. Patients with complete brain relaxation do not require any further medical management and the bone flap can be placed comfortably. However, patients with residual brain edema with or without secondary hydrocephalus and subdural fluid collection require further treatment measures. All those patients receive IV mannitol 1g/kg intraoperatively. If adequate brain relaxation is not achieved to place the bone flap comfortably, the bone flap is vertically cut into two pieces that are slightly separated and re-connected with a titanium mesh as seen in Figure 4. This adjustment provides an adequate increase in the size and flexibility of the flap to be placed over the defect without the need to apply pressure on the brain. This technique can also be applied in patients with hydrocephalus ex vacuo or subdural fluid collection when draining the excess fluid using an external ventricular drain, lumbar drain, or US needle tap fails to provide adequate brain relaxation.

**Figure 5:**
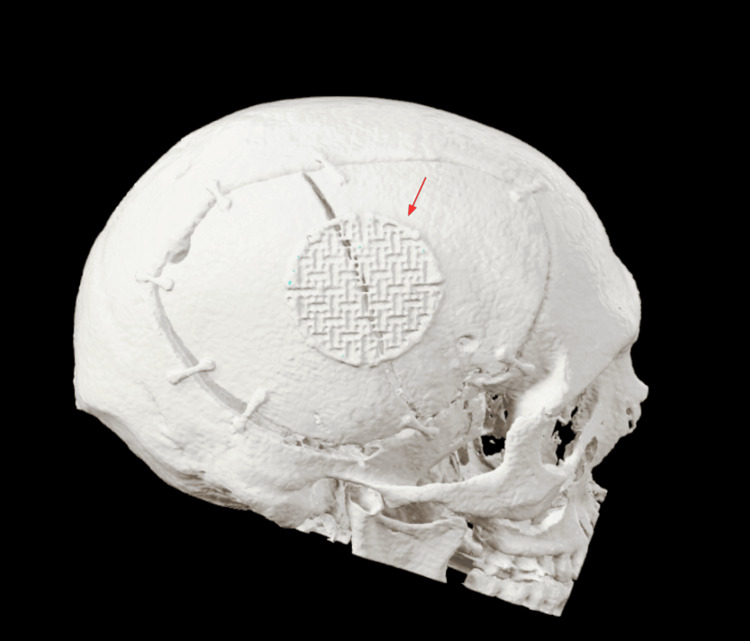
3-Dimensional representation of the technique to visualize the plates and hinge of the skull flap after placement.

Three patients consented to the procedure and underwent this operative technique due to persistent brain edema following DHC. All patients were admitted to the hospital postoperatively for observation and a CT head was routinely performed, which showed no complications. A 3-dimensional recreation of one of the scans is seen in Figure 5.

Patients were discharged on postoperative day one. At a four-week follow-up, all patients had a stable baseline functional exam with no visible cranial defect, complications, or wound issues. The patient information is displayed in Table 1.

**Table 1:** Patient information and follow-up.

	Sex/Age	Clinical History	Cerebral Edema/Previous Treatments	Time from Decompressive Hemicraniectomy to Cranioplasty (days)	Follow-up
Patient 1	M, 60	Right-sided: Internal Carotid Artery, Anterior Cerebral Artery, Middle Cerebral Artery Strokes. Malignant edema and brain shift	Brain swelling prevented flush placement of bone flap.	151	4 weeks – incision healing well and no cranial defect
Patient 2	F, 39	Left-sided: Middle Cerebral Artery Stroke. Malignant edema and brain shift associated with declining mental status.	Ventriculostomy to attempt to reduce swelling.	352	4 weeks – incision healing well and no cranial defect. Patient still has global aphasia from the stroke
Patient 3	F, 51	Right-sided; Intraparenchymal Hemorrhage	Intracranial cysts evacuated	240	4 weeks – incision healing well and no cranial defect. Remains non-ambulatory with reported left-sided spasms

## Discussion

Cranioplasty has different supporting evidence for optimal timing to reduce postoperative complications, but the data within the literature is conflicting on optimal timing [20]. Large-scale studies have shown an increased risk of hydrocephalus within 90 days, while waiting past this point increases the risk for new-onset seizures [21]. A systematic review involving 1209 patients assessing risk factors of early (one to three months) versus delayed (three to six months) cranioplasty found that both groups are comparable on rates of infection, subdural hematoma, and subdural fluid collection; however, postoperative rates of hydrocephalus were higher in the early cranioplasty groups [22]. Some data suggests that neurologic outcomes are better following early cranioplasty; however, more data is needed to support those findings [23]. While some studies have shown improved motor scores if cranioplasty is performed within 90 days, the mini-mental status exams and memory function were not significantly different for earlier operations [24]. When deciding to proceed with cranioplasty in patients with persistent cerebral edema, neurosurgeons commonly proceed with external ventricular drain or lumbar drain to reduce the swelling. However, those procedures could be associated with intracranial and tract hemorrhage, ventriculitis, technical failures such as inability to tap ventricle or misplacement, increased cost, problems from over-drainage, inadvertent vascular injury, pneumocephalus and pneumoventriculi, and CSF leak [12,25].

In this study we report a novel technique for two-piece cranioplasty that can be used in cases with lingering cerebral edema, preventing comfortable placement of the bone flap. This technique adequately increases the size and flexibility of the bone flap to be placed without applying pressure on the brain and could prevent the risks of prolonging cranioplasty or placing a drain.

The technique itself requires taking the existing bone flap and making a midline incision from a superior to inferior direction to create the shortest incision in the flap. This cut allows for an easier ability to limit a cranial defect and follow the general contour of the skull. In contrast, a longitudinal skull has more room to have an obvious defect and also fits less easily with the shape of the skull. Once the cut has been made, both pieces can be placed on the cranium to identify the angle needed to enclose the cortex. Subsequently, a nonabsorbable plate is positioned over the midline of the incision in the bone flap. Two hole cranial plates are placed on the periphery of the bone flap on both sections to lock it into the cranium with appropriate screws. Once the flap is soundly locked into place, the gala is closed in standard fashion, the skin is repaired with 2-0 Vicryl sutures, and staples are placed to reapproximate the scalp. Depending on the extent of the hinge angle, cranial defects can be avoided and are non-observable as long as the angle of midline placement is not too drastic.

This surgical technique's limitations primarily pertain to its usage as an adaptation of a normal cranioplasty but only in the presence of persistent swelling. This technique is not meant to replace a normal cranioplasty procedure but rather allow neurosurgeons to complete the surgery even with persistent cerebral swelling. Furthermore, this surgical technique has not been studied for its long-term effects on bone resorption or any other complications from altering the flap. Long-term patient follow-ups were not assessed as these cases were descriptors of the surgical technique employed.

Consequently, many risks must be weighed when deciding to proceed with cranioplasty; however, once the decision to operate is maintained, this decision brings on many costs for the patient such as an extended hospital stay. Foremost, patients must stop anticoagulants before undergoing the operation and endure a significant operation to replace the skull flap. If edema is too persistent despite additional measurements, the patient would have to be reclosed and undergo the process again. Moreover, these patients are already severely ill, so enduring general anesthesia and medication changes can be costly to the patient. This operative technique prevents halting anticoagulants, saves costs on hospital stays, plus aids patients in preventing unnecessary additional medications or surgeries. While debate remains about the optimal timing of cranioplasty, the decision to undergo the operation ideally occurs once. This operative technique allows neurosurgeons to perform a successful cranioplasty without applying unnecessary force to the cortex. Importantly, the decision to operate is already a risk in and of itself for these patients who are critically ill, so limiting it to one operating time can also save family and caregivers money and time in caring for the patient. Patients would have to remain in the hospital for shorter amounts of time and families would only need to commute the one time as well. If all the risk has been taken to operate on the patient initially, then this operative technique assures that the patient will only undergo one surgical event to place the bone flap.

## Conclusions

In this study, we present a novel technique that facilitates bone flap replacement during cranioplasty in cases with persistent brain edema without the need for invasive drain placement. The utilization of this technique permits neurosurgeons to complete the operation despite persistent cerebral edema. With an incision down the midline, the skull flap can be plated together at an angle to form an extra pocket of space to accommodate the swollen brain. By utilizing this technique, a neurosurgeon could prevent the cost and health risks of undergoing another operation for patients. All patients performed well with this alteration to the cranioplasty and did not have any changes to their recovery. The patients also did not exhibit any visible cranial defects on follow-ups. This technique can adequately reapproximate a skull flap and could prevent the need for a second operation.
